# The association between living alone and depressive symptoms and the role of pet ownership among Japanese workers

**DOI:** 10.1186/s12889-023-16619-2

**Published:** 2023-09-11

**Authors:** Haruka Miyake, Yosuke Inoue, Hiroko Okazaki, Toshiaki Miyamoto, Masafumi Eguchi, Takeshi Kochi, Isamu Kabe, Aki Tomizawa, Ami Fukunaga, Shohei Yamamoto, Maki Konishi, Seitaro Dohi, Tetsuya Mizoue

**Affiliations:** 1 Department of Epidemiology and Prevention, Center for Clinical Sciences, National Center for Global Health and Medicine, 1-21-1 Toyama, Shinjuku-ku, Tokyo, Japan; 2 Mitsui Chemicals, Inc, Tokyo, Japan; 3 Nippon Steel Corporation, East Nippon Works, Kimitsu Area, Chiba, Japan; 4 Furukawa Electric Co., Ltd, Tokyo, Japan; 5 KUBOTA Corporation Co., Ltd, Ibaraki, Japan; 6 Health Design Inc, Tokyo, Japan

**Keywords:** Pet ownership, Living alone, Depression, Depressive symptoms, CES-D, Japan

## Abstract

**Background:**

Living alone has been positively associated with the prevalence of depressive symptoms. We examined how a combination of living alone and pet ownership relates to depressive symptoms.

**Methods:**

As part of the Japan Epidemiology Collaboration on Occupational Health Study, we conducted a survey on health-related lifestyles, including living arrangements and pet ownership, among 12,763 employees of five companies in 2018–2021. Depressive symptoms were assessed using the 11-item Center for Epidemiological Studies-Depression Scale (cutoff score ≥ 9). A Poisson regression model with a robust variance estimator was used to calculate prevalence ratio and 95% confidence interval (CI) while adjusting for covariates.

**Results:**

Among the participants, 30.9% were depressed, 17.7% had pets, and 29.1% lived alone. Compared to individuals living with others but not with a pet, those living alone and not with a pet had a 1.17 times higher prevalence ratio of depressive symptoms (95% CI: 1.08-1.26). The corresponding figures were 1.03 (95% CI: 0.95-1.11) for those living with others and pet(s) and 1.42 (95% CI: 1.18-1.69) for those living alone but with pet(s).

**Conclusion:**

Living alone was significantly associated with a higher prevalence of depressive symptoms. The association was rather stronger among individuals with vs. without pets. Pet ownership may not be associated with decreased depressive symptoms.

**Supplementary Information:**

The online version contains supplementary material available at 10.1186/s12889-023-16619-2.

## Background

Globally, approximately 300 million adults experience depression [1]. In particular, the disease burden associated with depression is large among younger populations. In 2019, depressive symptoms were ranked sixth among the causes of disability-adjusted life years in the 25–49 age group [2]. Given that only one-third of patients diagnosed with depression achieve remission with the use of antidepressants, researchers and policy makers must address this global public health crisis and increase efforts to prevent the incidence of depression.

Living alone is considered a risk factor for depression. A meta-analysis of 16 studies by Hu et al. [3] reported that the odds ratio for having depressive symptoms was 1.44 times among those living alone vs. those living with others, suggesting that there is a relationship between living arrangements and depressive disorders. A more recent study by Kim et al. [4] using data collected from 4,223 individuals aged 65 years and older in Korea reported that those living alone had higher levels of depressive symptoms than those living with others. Similarly, a study by Pulkki-Råback et al. [5] that used data collected from 3,471 individuals in Finland (mean age: 44.6 years old) reported that those who lived alone were 1.81 times more likely to purchase antidepressants than those who did not live alone.

This study extends the previous research on the association between living alone and depressive symptoms by considering the role of pet ownership in this association. Previous studies have suggested that pet ownership may exert a protective effect against a worsening of mental health [6–8], albeit with some studies reporting null results [9]. For example, in their study of 823 residents aged 21–64 years in Singapore, Goh et al. [6] reported that pet owners who had primary caretaking responsibilities for pets had a significantly higher score in the emotional well-being domain of the Medical Outcomes Study 36-item short-form health survey (SF-36) compared to non-pet owners although no significant difference was found between pet owners and non-pet owners. Another study conducted by Hajek et al. [7] among 1,160 older adults in Germany reported that dog owners were less socially isolated compared to individuals without pets. Examining the association between living alone, pet ownership, and depressive symptoms is particularly important in a society where the number of individuals living alone and those with depressive symptoms are both increasing.

Therefore, this study explored the association between living alone and depressive symptoms among workers in Japan and examined this association considering the role of pet ownership. We hypothesized that the positive association between living alone and depressive symptoms was attenuated among those living with pets. Given the epidemiological data suggesting an increase in the risk of suicide associated with other forms of family structure (e.g., living only with parents [10]), we also examined depressive symptoms in relation to the combination of living arrangements and pet ownership.

## Methods

### Study design

As part of a large cohort study (the Japan Epidemiology Collaboration on Occupational Health [J-ECOH] Study [11–13], a self-administered questionnaire survey was conducted at five participating companies of the J-ECOH via a paper-based questionnaire (three companies) or a web-based questionnaire (two companies) between April 2018 and February 2021. In the questionnaire, we asked about health-related lifestyles (including pet ownership and living arrangements) and depressive symptoms. We also obtained the data of annual health checkups that were conducted by the employers during the same fiscal year as the questionnaire survey.

We explained the objective of the survey in the first page (for the paper-based questionnaire) or window (for the online questionnaire) of the questionnaire. Consent was confirmed if a potential participant signed and returned the paper-based questionnaire or clicked the consent box of and completed the online questionnaire.

### Study participants

Among the 17,078 workers whose health checkup data was available, 12,847 participated in the questionnaire survey (response rate: 75.2%). We excluded those with missing information about pet ownership, depressive symptoms, or covariates (*n* = 84), leaving 12,763 participants in the main analysis.

### Outcome

Depressive symptoms were assessed using the 11-item Center for Epidemiological Studies - Depression Scale (CES-D-11). Participants were asked to respond to questions on 11 symptoms associated with depressive disorder that participants had experienced in the previous week with four response options including “rarely or none of the time (< 1 day)”; “some or a little of the time (1–2 days)”; “occasionally or a moderate amount of the time (3–4 days)”; and “most or all of the time (≥ 5 days)”. After reverse scoring two positive questions (“I enjoy every day” and “I can spend my time being satisfied about life”), we assigned a score ranging from 0 to 3 to each response and calculated the total score (0–33). The Cronbach’s alpha was 0.80 in this study. Then in accordance with previous studies [14–17], a score of ≥ 9 was defined as having depressive symptoms.

### Exposure

We asked participants whether they lived with one or more of the following (i.e., allowing multiple responses): spouse (wife/husband); child(ren) of preschool age; child(ren) of primary school age or older; parent(s); sibling(s); grandparent(s); pet(s); and other(s). Those who answered in the negative to all the options except for that of a pet were defined as those living alone while those who answered in the affirmative to the question regarding pet ownership were defined as those with a pet. Based on these two variables (i.e., living alone and pet ownership), the participants were classified according to the following four categories: (1) living alone with pet(s); (2) living alone without a pet; (3) living with others and pet(s); and (4) living with others but without a pet.

### Covariates

Information on sociodemographic variables was obtained via the questionnaires, including: age (in years); sex (male; female); marital status (married; not married); employment status (upper management; middle management; other); and educational background (9–12 years; 13–16 years; ≥ 17 years).

We also collected questionnaire information on lifestyle parameters that are known to be associated with depression/depressive symptoms (i.e., smoking and alcohol consumption) [18, 19]. Specifically, we asked the participants their smoking status and categorized them into the following groups, i.e., never, former, and current smoker. Alcohol consumption was estimated based on the questionnaire information on consumption frequency (i.e., never drink/quit; 1–3 days/month; 1–2 days/week; 3–4 days/week: 5–6 days/week; daily) and amount consumed per occasion (i.e., 0.5 *go*; 1 *go*; 1.5 *go*; 2 *go*; 2.5 *go*; 3 *go*; 3.5 *go*; 4 *go* or more; 1 *go* is the traditional Japanese unit and is equivalent to approximately 20 g of ethanol). We assigned the values to each response option for consumption frequency ranging from never drink/quit = 0; 1–3 days per month = 0.5; 1–2 days per week = 1.5; 3–4 days per week = 3.5; 5–6 days per week = 5.5; to daily = 7, which were then multiplied with the amount to compute daily alcohol consumption. The participants were categorized according to the following groups: did not drink, drank < 1 *go*/day; drank 1–1.9 *go*/day; drank ≥ 2 *go*/day.

### Statistical analysis

To examine the association of the combinations of living arrangements and pet ownership in relation to depressive symptoms, we ran a Poisson regression analysis with a robust variance estimator to estimate prevalence ratios (PRs) and corresponding 95% confidence intervals (CIs) while adjusting for sociodemographic and lifestyle-related variables and study site (five sites) [20, 21]. We confirmed that there was no evidence of multicollinearity (i.e., the variance inflation factor was 1.88).

In accordance with a previous study in Japan that examined the association between living arrangements and the risk of suicide, and found an elevated risk of suicide even among those who did not live alone [10], we categorized participants according to 16 categories defined by the combinations of living arrangements, that is, living with spouse, children, and birth family (i.e., biological parents or siblings or grandparents) and pet ownership, and conducted a sensitivity analysis. Those who responded that they lived with other(s) but without specific details were excluded from this analysis (*n* = 217), resulting in an analytic sample of 12,569 participants.

Statistical analysis was conducted using the Stata® Version 16.0 statistical software package (StataCorp., College Station, Texas, USA).

## Results

Table 1 shows the basic characteristics of the study participants by the combination of living alone status and pet ownership. The mean age was 42.5 years (standard deviation [*SD*] = 12.4) and females comprised 12.1% of the study participants. The proportion of those who were married and in upper or middle management positions were higher among those who lived with others vs. those who lived alone. Those who had never smoked comprised 49% of study participants, with the highest figure reported among those who did not live with others or pet(s) (60.9%). Those who did not live with a pet tended to have higher educational levels than those who did.

**Table 1:** Basic characteristics of study participants

	All participants (*n* = 12,763)		The combinations of living alone status and pet ownership							
			Living alone without a pet (*n* = 3559)		Living alone with pet(s) (*n* = 154)		Living with others but without a pet (*n* = 6945)		Living with others and with pet(s) (*n* = 2105)	
Age, mean [*SD*]^a^	42.5	[12.4]	35.3	[12.9]	43.8	[12.9]	44.7	[11.0]	47.1	[10.6]
Female, *n* (%)	1540	(12.1)	456	(12.8)	30	(19.5)	813	(11.7)	241	(11.4)
Marital status, *n* (%)	8168	(64.0)	412	(11.6)	52	(33.8)	5918	(85.2)	1786	(84.8)
Employment status, *n* (%)										
Upper management	563	(4.4)	94	(2.6)	4	(2.6)	352	(5.1)	113	(5.4)
Middle management	3477	(27.2)	557	(15.7)	26	(16.9)	2238	(32.2)	656	(31.2)
Other	8723	(68.3)	2908	(81.7)	124	(80.5)	4355	(62.7)	1336	(63.5)
Alcohol consumption, *n* (%)										
Don’t drink	3232	(25.3)	1002	(28.2)	44	(28.6)	1668	(24.0)	518	(24.6)
< 1 *go*^b^	6179	(48.4)	1905	(53.5)	77	(50.0)	3316	(47.7)	881	(41.9)
1–1.9 *go*^b^	2045	(16.0)	383	(10.8)	22	(14.3)	1225	(17.6)	415	(19.7)
≥ 2 *go*^b^	1307	(10.2)	269	(7.6)	11	(7.1)	736	(10.6)	291	(13.8)
Smoking status, *n* (%)										
Never	6252	(49.0)	2166	(60.9)	74	(48.1)	3258	(46.9)	754	(35.8)
Former	3821	(29.9)	621	(17.4)	48	(31.2)	2351	(33.9)	801	(38.1)
Current	2690	(21.1)	772	(21.7)	32	(20.8)	1336	(19.2)	550	(26.1)
Educational background, *n* (%)										
9–12 years	6358	(49.8)	1704	(47.9)	94	(61.0)	3323	(47.8)	1237	(58.8)
13–16 years	3503	(27.4)	905	(25.4)	39	(25.3)	1976	(28.5)	583	(27.7)
≥ 17 years	2902	(22.7)	950	(26.7)	21	(13.6)	1646	(23.7)	285	(13.5)

^a^*SD* standard deviation

^b^*go*: 1 *go* is the Japanese traditional unit equivalent to approximately 20 g of ethanol

Table 2 shows the results of the Poisson regression analysis with a robust variance estimator examining the association between living arrangements, pet ownership, and depressive symptoms. Compared to those who lived with others, those who lived alone were more likely to report depressive symptoms (PR = 1.17, 95% CI: 1.09–1.26) (Model 1). In Model 2 in which we examined the role of pet ownership in the association between living alone and depressive symptoms, the prevalence of depressive symptoms was higher in those living alone without a pet (PR = 1.17, 95% CI: 1.08-1.26) and those living alone and with a pet (PR = 1.42, 95% CI: 1.18–1.69), compared to those who lived with others but not with a pet. When we examined the association between pet ownership and depressive symptom, we did not find any evidence of significant association (PR = 1.04, 95% CI: 0.97–1.11) (data not shown in table).

**Table 2:** Result of a poisson regression model with a robust variance estimator examining the cross-sectional associations between living alone status and depressive symptoms among 12,763 study participants in Japan (2018–2021)

	Number of participants with/without depressive symptoms	PR^a^ (95% CI)
**Model 1**		
Not living alone	2457 / 6593	1.00 (Reference)
Living alone	1482 / 2231	1.17 (1.09–1.26)
**Model 2**		
Living with others but without a pet	1869 / 5076	1.00 (Reference)
Living with others and pet(s)	588 / 1517	1.03 (0.95–1.11)
Living alone without a pet	1414 / 2145	1.17 (1.08–1.26)
Living alone with pet(s)	68 / 86	1.42 (1.18–1.69)

Adjusted for age, sex, marital status, employment status, alcohol consumption, smoking status, educational background, and study site

^a^*PR* prevalence ratios

Supplementary Table 1 shows the results of the sensitivity analysis in which we further categorized participants according to 16 groups defined by living arrangement categories and pet ownership. Compared to those who lived with a spouse and children, the prevalence of depressive symptoms was higher among those living alone (PR = 1.33, 95% CI: 1.16-1.52), living alone but with a pet (PR = 1.55, 95% CI: 1.27-1.91), living with their birth family only (PR = 1.21, 95% CI: 1.03-1.43), and living with their birth family and pet(s) (PR = 1.31, 95% CI: 1.07-1.60). When we compared the effect sizes reported for any combinations of exposures that differed only in terms of pet ownership, we did not find any evidence of lower depressive symptoms among pet owners.

## Discussion

Among 12,763 Japanese workers, we found that living alone was positively associated with the prevalence of depressive symptoms. When we examined the associations in relation to the combinations of living alone status and pet ownership, we found that those living alone without a pet and those living alone but with a pet were both associated with a higher prevalence of depressive symptoms, with a stronger association found among those living alone with vs. without a pet.

Our findings suggesting a higher prevalence of depressive symptoms among those living alone is in accordance with the findings reported in the meta-analysis by Hu et al. [3], that reported that the odds ratio for having depressive symptoms was 1.44 times among individuals living alone vs. those living with others. Although the majority of the extant literature examined the association among older individuals [3, 4], we extended those studies by showing the association between living alone and depressive symptoms among relatively young employees. Although these employees may have spent a significant amount of time interacting with coworkers, further research is needed to confirm the relationship between living alone and negative health outcomes.

When we examined the associations in relation to the combinations of living alone status and pet ownership, the associations were stronger among those living alone but with pets vs. among those living alone and without pets. This was not in accordance with the findings reported by Hajek et al. [7], that showed that dog owners were less likely to report social isolation compared to individuals without pets. It also contrasts with the results of the study of Goh et al. [6], that demonstrated that pet owners who had primary caretaking responsibilities for their pets had a significantly higher score in the emotional well-being domain of the SF-36 compared to that of non-pet owners. Whereas we formulated our hypothesis based on these previous studies that suggested the possible protective effect of pet ownership, our hypothesis was not supported in this study.

There are several possible interpretations for our null findings. First, despite the psychological benefits of pet ownership as demonstrated by previous studies, pet ownership may also be associated with some forms of burden. An online survey of 1,017 pet owners aged 18–76 years in the United States [22] reported that 75% of pet owners said that cost of pet care is rising, and they are struggling to afford it. Second, it is possible that pet owners may experience sleep deprivation as a result of their pets walking around in the room or meowing to demand food during nighttime. A previous study conducted by Van Egmond et al. [23] reported that the odds ratio for not achieving the recommended seven hours of sleep per day are 1.18 times among cat owners vs. non-cat owners (95% CI: 1.02-1.37). Although the study participants were highly selective (i.e., pregnant mothers) and the outcome was slightly different (i.e., postpartum depression), Matsumura et al. [24] suggested that cat ownership may cause a worsening of mental health among their study’s 80,814 female participants in Japan.

When we further categorized the participants according to living arrangement categories and examined their association with depressive symptoms, we found that the prevalence of depressive symptoms was higher among those who lived only with their birth family, compared to those who lived with a spouse and children. This result is in accordance with the results of Poudel-Tandukar et al.’s study [10], that found that suicide risk was elevated not only among those living alone, but also among those living only with parents, using data collected from individuals who participated in the Japan Public Health Center Study. In our study, a protective effect of pet ownership was not observed among those who lived with their birth family, either.

This study has several limitations. First, regarding employment, the study’s participants were employed by relatively large companies in Japan and may not have been fully representative of the Japanese working population. It is also possible that time spent with pets would be different between those who were currently employed and who were unemployed. In addition, the higher proportion of male participants in this study presents a possible threat to generalizability. Second, this study did not provide information on the type of pet or whether the participants were pet owners with primary caretaking responsibilities of their pets. Previous studies [6, 7] have found significant associations with mental health among dog owners and those pet owners who were the primary caretakers of a pet. It is possible that a statistically significant association could have been observed in our study had we had that information. Third, the study’s cross-sectional design does not allow us to infer a causal relationship of the association observed in this study. For example, it is possible that those who experience depressive feelings and live alone want to own a pet to relieve their loneliness. In fact, Sharpley et al. [9] examined the cross-sectional association between CES-D scores and pet ownership and demonstrated that a 1-unit increase in the score was associated with a 7% increase in the probability of owning a dog. Finally, we used the CES-D-11 scale to assess depressive symptoms and did not include the category of clinical depression.

## Conclusion

In conclusion, we found that living alone was associated with the prevalence of depressive symptoms among 12,763 Japanese workers. When we examined the association of the combination of living alone status and pet ownership, we did not observe significant association between pet ownership and depressive symptoms. Further investigation is required to examine how to protect individuals who live alone.

### Supplementary information

**Additional file 1.**

## Data Availability

The data are not publicly available but are available upon reasonable request to the corresponding author.

## Abbreviations

CES-D-11: 11-Item Center for Epidemiological Studies - Depression Scale
PR: Prevalence ratio
CI: Confidence interval
SD: Standard deviation
