# Supplementary material for: The association between living alone and depressive symptoms and the role of pet ownership among Japanese workers
Source: BMC Public Health. 2023 Sep 11;23:1769. doi: 10.1186/s12889-023-16619-2 (PMC10494449; doi:10.1186/s12889-023-16619-2)
Supplement: Supplementary file 1 — Additional file 1. [file 12889_2023_16619_MOESM1_ESM.docx]

**Supplementary Table 1**

Result of a Poisson regression model with a robust variance estimator examining the cross-sectional associations between living arrangements and depressive symptoms among 12,569 study participants in Japan (2018–2021).

| Living arrangements | Number of participants with/without depressive symptoms | PR^1^ | 95%CI |
| --- | --- | --- | --- |
| Spouse + Child(ren) | 930 / 2786 | 1.00 | (reference) |
| Alone | 1414 / 2145 | 1.33 | (1.16-1.52) |
| Pet(s) only | 68 / 86 | 1.55 | (1.27-1.91) |
| Child(ren) only | 59 / 143 | 1.11 | (0.89-1.39) |
| Child(ren) + Pet(s) | 15 / 44 | 0.91 | (0.58-1.42) |
| Birth family only | 286 / 495 | 1.21 | (1.03-1.43) |
| Birth family + Pet(s) | 101 / 140 | 1.31 | (1.07-1.60) |
| Birth family + Child(ren) | 10 / 26 | 0.98 | (0.57-1.68) |
| Birth family + Child(ren) + Pet(s) | 5 / 12 | 0.99 | (0.47-2.06) |
| Spouse only | 416 / 1201 | 1.01 | (0.91-1.11) |
| Spouse + Pet(s) | 127 / 370 | 1.02 | (0.87-1.19) |
| Spouse + Child(ren) + Pet(s) | 264 / 728 | 1.06 | (0.95-1.19) |
| Spouse + Birth family | 27 / 75 | 1.06 | (0.77-1.47) |
| Spouse + Birth family + Pet(s) | 18 / 63 | 0.87 | (0.58-1.32) |
| Spouse + Birth family + Child(ren) | 84 / 232 | 1.01 | (0.84-1.23) |
| Spouse + Birth family + Child(ren) + Pet(s) | 47 / 130 | 1.00 | (0.78-1.29) |

^1^PR= prevalence ratios

Adjusted for age, sex, marital status, employment status, alcohol consumption, smoking status and educational background.

Those who responded that they lived with “other(s)” were excluded from this analysis (*n* = 217).
